# Melody transposition tolerance in the human cortex: An fMRI adaptation and MVPA investigation

**DOI:** 10.1162/imag_a_00352

**Published:** 2024-11-01

**Authors:** Yun-An Han, Po-Jang Hsieh

**Affiliations:** Department of Psychology, National Taiwan University, Taipei, Taiwan

**Keywords:** music perception, music cognitive neuroscience, fMRI, fMRI adaptation, multivariate cross-classification, melody perception

## Abstract

Melody perception involves constant relational representations, enabling most people to recognize the melodies after being transposed. This invariant property of melody transposition has been supported in many previous behavioral studies, and we hypothesize that there are brain regions showing tolerance toward melody transposition when processing melodies. To test the hypothesis, we adopted an event-related adaptation approach and a multivariate pattern cross-classification (MVCC) analysis approach. Consistent with our prediction, we discovered clusters in the right superior temporal gyrus (STG) and the left middle temporal gyrus (MTG) that exhibited adaptation when participants listened to both the same and transposed-same melodies after the original ones. An ROI and searchlight-based cross-classification analysis also revealed that BOLD pattern in the bilateral precentral gyrus (PreCG), the left inferior frontal gyrus (IFG), the left inferior parietal lobule (IPL), the right angular gyrus (AG), and the right superior temporal gyrus (STG) showed tolerance to melody transposition. These findings suggest that tolerance to melody transposition exists throughout the music processing pathway from auditory to motor cortices.

## 1 Introduction

People from various cultures interact with music in many ways, such as listening to, remembering, or creating melodies. Some melodies, like the well-known “Happy Birthday” song, are frequently used or sung. Interestingly, even though people can sing “Happy Birthday” together at celebrations, there are differences in the pitch, tone, and key when different individuals recall or perform it. This variation occurs because, when people remember a melody, they recall the consistent pitch relationships within the melody, but not necessarily the exact pitches.

### 1.2 Two-component theory of melody perception and behavioral and neural evidences of the invariance of melody transposition

Dowling (1978)proposed the “Two-Component Theory” of melody, suggesting that melody perception consists of two components. The first one is “melodic contour”, the pattern of pitches moving up or down within the melody. The second component is the more precise distances between the notes that make up the melodic contour, known as “musical intervals/scales.” These distances are typically measured in semitones or whole tones in the framework of Western music theory.

Both “contour” and “intervals” in a melody are contingent upon the “relative” relationships between notes, rendering them unaffected by changes in “absolute” pitch when transposed. Transposition involves relocating melodies to a different key while preserving their contour and intervals. Most individuals, irrespective of their musical expertise, can readily identify or even memorize well-known melodies, such as the “Happy Birthday” song, even when transposed, underscoring the invariant nature of melody transposition.

Previous behavioral research has shed light on the existing transposition invariance in melody perception. Whether transposed or not, the consistent frequency relations among pitches constitute the fundamental perceptual feature of melodies (Croonen & Kop, 1989;Dowling, 1978;Dowling & Bartlett, 1981;Dowling & Fujitani, 1971;Edworthy, 1985). Even infants as young as six months exhibit this perceptual invariance (Plantinga & Trainor, 2005). Transposed versions of familiar melodies do not capture their attention, indicating that infants can memorize melodies based on their relative pitch profile and consider the transposed versions as the same as the original ones.

Despite robust behavioral evidences supporting melody transposition invariance, research on the neural underpinnings of this phenomenon remains limited.Schindler et al. (2013)utilized fMRI to investigate whether melody invariance (“Gestalt”) is represented in the early auditory cortex. Employing multivariate pattern analysis (MVPA), they discovered that patterns of BOLD activation in bilateral Heschl’s gyrus (HG) and planum temporale (PT) accurately classified transposed melodies based on their original versions. Another fMRI study byLee et al. (2011)revealed that brain activation patterns in the right superior temporal sulcus (STS), left inferior parietal lobule (IPL), and anterior cingulate cortex (ACC) could significantly classify ascending and descending contours, suggesting that these brain regions were responsible for melodic contour processing. Since the contours remained intact after transposition, these contour processing regions may be tolerant to melody transposition.

### 1.3 The influences of absolute pitch ability and musical expertise

While transposed melody perception exhibits invariance across most individuals, two factors—individuals’ absolute pitch (AP) ability and their musical training experiences—can influence this phenomenon. It is crucial to recognize that when a melody is transposed, the absolute pitches within the melody are altered.Miyazaki (2004)found that musicians with the ability to label absolute pitch performed less effectively when discriminating transposed melodies. While he suggested that AP ability might hinder relative pitch capacity, an alternative explanation posits that AP possessors may employ inappropriate absolute pitch strategies for the given task (Aruffo et al., 2014). In either case, AP ability has the potential to interfere with the invariance of melody transposition to some extent.

Participants’ musical backgrounds also introduce a potential confounding factor in the perception of transposed melodies. Although most individuals can readily recognize a transposed melody regardless of their musical experience, a study byFujioka et al. (2004)revealed that pitch interval and melody contour deviations elicited more pronounced MMNm responses in musicians compared to non-musicians, where MMNm responses were nearly absent. These findings deviated from previous research (Trainor et al., 2002), with the inconsistency attributed to the poorer behavioral performance of non-musicians in 2004. Additionally, other evidence suggested that musicians outperformed non-musicians in transposed melody discrimination tasks, with musical training accounting for over 68% of the variance (Foster & Zatorre, 2010). Consequently, individuals’ experiences of musical training may more or less interfere with the invariant property of melody transposition. We controlled both the participants’ AP abilities and musical background to ensure that the results of ours can be generalized to most individuals.

### 1.4 The purposes of current study

The main purpose of the current study was to (1) find the regions tolerant to melody transposition with two different approaches: an fMRI adaptation approach and a multivariate pattern analysis (MVPA) approach, and (2) investigate whether the behavioral performance of the transposed melody discrimination was related to the neural tolerance to melody transposition.

#### 1.4.1 Adaptation approach

First, to pinpoint the brain regions that exhibit the invariance of melody transposition, we utilized an event-related fMRI adaptation paradigm inspired byDilks et al. (2011). fMRI adaptation paradigm was initially developed byGrill-Spector and Malach (2001)as a tool for studying the functional invariant properties of human cortical neurons. This approach reflects the fact that neural activity related to successive stimuli will get smaller if they activated the same pool of neurons, and can thus be used to identify those brain regions in which successive stimuli share a common neural representation. The method was not only applied in the visual domain (e.g.,Dilks et al., 2011;Grill-Spector & Malach, 2001), but also utilized to inspect the auditory invariant properties such as lyrics or tunes in music (Sammler et al., 2010) or categorical processing in speech (Raizada & Poldrack, 2007). To investigate melody transposition invariance, participants engaged in a melody discrimination task, adapted fromFoster & Zatorre (2010), requiring them to discern whether two consecutively presented melodies were identical or different, irrespective of transposition. Our aim was to identify regions exhibiting tolerance to melody transposition, positing that the regions of interest would manifest adaptation solely when exposed to successions of transposed-same and non-transposed-same melodies rather than those with deviant intervals.

#### 1.4.2 MVPA approach

Second, to determine whether the brain can tolerate the transposition of melodies during their processing, specifically whether melody perception remains unaffected by transposition, we conducted a multivariate cross-classification (MVCC) analysis, a method that involves training a classifier on data from one cognitive domain or experimental condition and testing it on data from another, thereby revealing patterns indicative of invariance across contexts (Kaplan et al., 2015). The MVCC method was employed to train classifiers to distinguish brain activation patterns when subjects heard the same vs. different melodies. In brain regions where these conditions could be distinguished, further tests were conducted to determine if the same distinctions could be made under conditions of transposition. We incorporated both a predefined region of interest (ROI) analysis and an additional exploratory whole-brain searchlight analysis.

#### 1.4.3 The relationships with behavioral performance

The other objective of the research was to investigate the relationship between individuals’ behavioral relative pitch (RP) abilities in the context of transposed melody discrimination and their neural tolerance to melody transposition. We sought to determine whether individuals with stronger RP abilities were more inclined to perceive transposed and non-transposed melodies as identical. To assess this hypothesis, we computed the accuracy of transposed conditions, providing valuable insights into participants’ RP abilities based on their performance in transposed melody discrimination. Importantly, to control for the influence of AP abilities and musical training, we recruited participants with and without professional musical training backgrounds, and evaluated their AP abilities using a tone-naming test.

## 2 Methods

### 2.1 Participants

We recruited 22 healthy volunteers with normal hearing and sight. Each participant provided self-reported information on their musical experiences through a questionnaire, which included inquiries about their professional musical training, involvement in amateur musical activities, and personal hobbies related to music listening. Notably, none of the participants were professional musicians or music major students. The criterion for “professional musical training (PMT)” was whether individuals had received one-on-one formal musical or instrument lessons, or if they had attended any officially recognized music talent classes in the past. Based on this PMT information, participants were categorized into two groups, as outlined inTable 1:11 individuals with amateur musical experience (Musicians) and 11 individuals without any musical background (Non-musicians). This study was approved by the ethics committee of the National Taiwan University, Taiwan.

**Table 1.** The mean characteristics of participants of musicians and non-musicians.

	Musicians	Non-musicians
Number of participants	11	11
Sex (female/male)	6/5	5/6
Handedness (right/left)	11/0	10/1
Age (years)	20.82	21.91
Years of musical training	6.91	0.00
Last time actively engaged in music (days before)	159.27	249.86
Estimates of hours of music listening per day	1.20	3.09

### 2.2 Tone-naming/AP test

Participants underwent a tone-naming assessment to evaluate their absolute pitch (AP) proficiency, as outlined by previous studies (Leipold et al., 2019;Oechslin et al., 2010). The subsequent melody discrimination task featured a pitch range spanning from E3 to C6, adhering to the 12-tone equal temperament tuning with A4 set at 440 Hz. To ensure equal exposure to each tone, every tone from #C3 to C6 was presented three times throughout the entire task. The task comprised three blocks, encompassing a total of 36 notes presented in a randomized sequence.

Each tone, lasting 1000 ms, was accompanied by 4000 ms of Brownian noise both preceding and following the tone. Participants were tasked with identifying the chroma of the tones within a four-second window after the tone presentation, disregarding the octave (Deutsch, 2013). Participants’ AP ability was quantified based on the accuracy of chroma identification.

All tones were generated with the piano timbre using MuseScore (https://musescore.com/). The assessment took place in a soundproof environment, with stimuli delivered through Sony headphones to minimize external interference. PsychoPy (https://www.psychopy.org/) was employed for stimulus presentation and response measurement.

### 2.3 Melody discrimination task

During fMRI scanning, participants engaged in a melody discrimination task adapted fromFoster and Zatorre (2010)andMiyazaki (2004). Each trial comprised two stimuli: an original melody and a probe melody. Participants were tasked with determining whether the probe melodies were identical to or different from the original ones. The stimuli, generated using MuseScore, were presented via PsychoPy through Sensimetric Model S14 insert headphones designed for fMRI research.

All melodies, featuring the piano timbre, initiated with the tonic note and consisted of seven eighth notes within the range from the lower fifth degree to the upper sixth degree note, along with an eighth rest. To maintain dynamism, successive intervals within the melodies were not uniformly large. Each note had a duration of 312.5 ms, equivalent to quarter notes at a tempo of 96 beats per minute, resulting in a total duration of 2.5 s for each melody.

Twenty-four original melodies were created to introduce diversity. Unlike prior research that predominantly used melodies in C Major, the present paradigm utilized four keys (A, C, E flat, F sharp major, six original melodies in each key) chosen for their identical pitch and harmonic distances on the circle of fifths. This choice aimed to control the pitch heights within the melodies when transposed. The starting notes were A3, C4, ♭E4, and ♯F4.

**Fig. 1.**
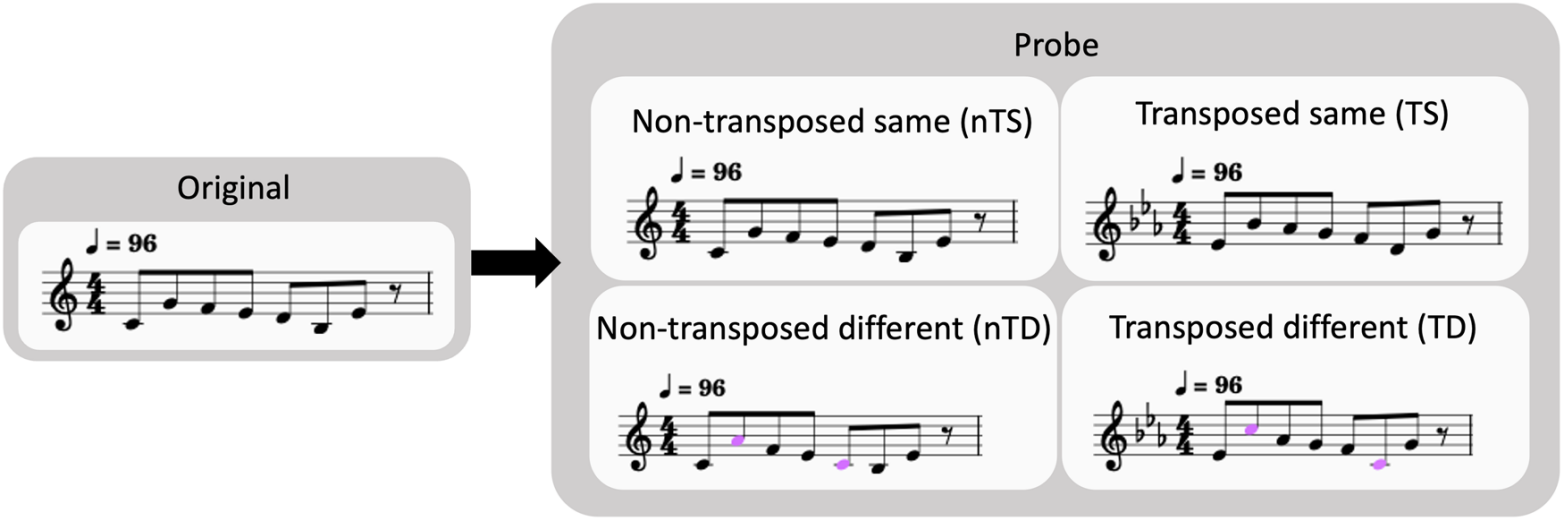
Examples of task stimuli.

Four probe conditions were designed: non-transposed same (nTS), non-transposed different (nTD), transposed same (TS), and transposed different (TD). In “different” trials, two of the middle five notes were shifted two semitones higher or lower to maintain the key and the contour of the melodies. To prevent participants from relying solely on melodic contour in the “different” trials, the contours were preserved (Bartlett & Dowling, 1980;Cuddy & Cohen, 1976;Trehub et al., 1993). In the “non-transposed” trials, probe melodies were in the same key as the originals, while in “transposed” trials, probe melodies were shifted to new keys by three different pitch distances: three, six, or nine semitones higher. The decision to emphasize “pitch” distance over “harmonic” distance followedKleinsmith & Neill’s (2018)suggestion that only pitch distance affects the discriminability of transposed melodies. All transpositions were upward to maintain tones within the range of E3 to C6.Figure 1is an example of the stimuli in all conditions. Participants were instructed to make judgments depending on the melody but not the pitch. We used clear illustration during the task instruction to ensure that they fully understood the task demands.

The in-scanner task followed a rapid event-related design, consisting of four counterbalanced runs and two pseudorandomized runs, each with 24 trials. Four counterbalanced runs comprised 96 unique pairs of the original melody and the probe condition, while two additional pseudorandomized runs with 48 out of those 96 pairs of trials were included to fill up the 1-hour scanning. In each trial, the original melody was presented first, followed by a 3.5 s interstimulus interval (ISI), and then the probe melody. Participants indicated their judgment using the right index or middle finger (counterbalanced) within 3.5 s. Fixation crosses were displayed during runs. Trial order and inter-trial intervals (ITI) in every run were optimized using the scheduling tool optseq2 (https://surfer.nmr.mgh.harvard.edu/optseq/), with ITIs jittered from 0 to 12 s for optimal efficiency.

### 2.4 fMRI data acquisition

Functional imaging was conducted on a 3T Siemens Magnetom Skyra at the Taiwan Mind & Brain Imaging Center. A 1 x 1 x 1-mm high-resolution T1-weighted MPRAGE anatomical scan was acquired before the functional runs. Functional MRI runs were scanned using an echo-planar imaging (EPI) sequence, and the parameters were as follows: TR = 2000 ms, TE = 24 ms, field of view = 220 x 220 mm, 38 slices per run, voxel size = 3.4 x 3.4 x 4.0 mm, and flip angle = 90^o^.

### 2.5 Behavioral data analysis

The behavioral results of the tone-naming test and in-scanner task were subjected to analysis using the statistical software R (http://www.r-project.org). In the case of the AP test, the average accuracy was computed to derive each participant’s AP score. Following the criteria set byAruffo et al. (2014), scores exceeding 0.9 were utilized to categorize participants as absolute pitch possessors.

For the melody discrimination task measures, a mixed-design ANOVA was conducted, incorporating a within-subject factor (condition: nTS, nTD, TS, TD) and a between-subject factor (group: musicians vs. non-musicians). Subsequently, the performance of transposed melody discrimination, defined by the average accuracy of transposed-same and transposed-different trials, was computed as participants’ RP performance to explore the relationship between participants’ RP and AP ability. A correlation analysis was then conducted between participants’ AP score and RP performance.

These analyses were undertaken to provide insights into the participants’ performance across different tasks and to examine potential associations between absolute and relative pitch abilities in the context of melody discrimination.

### 2.6 fMRI data preprocessing and first-level analysis

The functional and anatomical images underwent preprocessing and analysis utilizing SPM12 (http://www.fil.ion.ucl.ac.uk/spm/software/spm12). Following slice time and motion correction, individuals’ functional Echo-Planar Imaging (EPI) scans were co-registered with their T1 images and subsequently normalized to the Montreal Neurological Institute (MNI) template. For the univariate analysis, the normalized images underwent smoothing with an 8 mm full-width half-maximum Gaussian kernel. However, unsmoothed data were utilized for subsequent multivariate pattern analysis (MVPA).

A General Linear Model (GLM) design was established, encompassing eight task-related parameters (original and probe separately in four conditions, as outlined inTable 2), using boxcar functions convolved with a hemodynamic response function (HRF). In addition, six non-interested parameters accounting for head movements were included as covariates of no interest. This approach allowed for a comprehensive analysis of task-related activation and facilitated subsequent investigations into the neural underpinnings of different conditions.

**Table 2.** The 8 task-related regressors used in GLM design

Condition	Melody 1: Original	Melody 2: Probe
Non-transposed same (nTS/OS)	O _(prePos)_	Pos
Non-transposed different (nTD/OD)	O _(prePod)_	Pod
Transposed same (TS)	O _(prePts)_	Pts
Transposed different (TD)	O _(prePtd)_	Ptd

### 2.7 Univariate analysis (adaptation approach)

A whole-brain univariate analysis was performed to find the brain regions showing relatively more decrease of BOLD activation when the presented melodies following by the transposed-same and non-transposed-same versions than by the versions with deviant intervals. The term “adaptation” here refers to the repetitive suppression of brain activation, and was discerned by subtracting the probe from the original (e.g., [O_(prePos)_- Pos]). In the univariate analysis, only data from the four counterbalanced runs were considered to control for the equal probabilities of every combination of the original melody and the experimental condition. This approach aimed to pinpoint the repetitive suppression of BOLD activation that was specific to the melody itself but not any other auditory-related features. To achieve the goal, an “uninterested adaptation” mask was created at the group level using the combination of the adaptation of non-transposed different (nTD) and transposed different (TD), that is, (O_prePod_– Pod) + (O_prePtd_– Ptd), thresholded at*p*(unc.) < .05. Subsequently, the combination of the adaptation of non-transposed same (nTS) and transposed same (TS), that is, (O_prePos_– Pos) + (O_prePts_– Pts), were calculated, and examined at a threshold of*p*(unc.) < .001 and cluster size k >= 10 while exclusively masked by the “uninterested adaptation” mask. Surviving regions in this contrast would signify a “melody-specific adaptation effect” and indicate the representation of melody transposition invariance.

We extracted the parameter estimates within those surviving regions, and calculated the contrast of {[(O_(prePos)_- Pos) + (O_(prePts)_- Pts)]— [(O_(prePod)_- Pod) + (O_(prePtd)_- Ptd)]} to be the estimates of the “melody-specific adaptation effect”. Using the estimates, a subsequent correlation analysis explored the relationship between the melody-specific adaptation effect in these surviving regions and the behavioral performance of transposed melody discrimination to assess whether BOLD activation in these areas could predict behavioral performances.

### 2.8 Multivariate pattern analysis (MVPA)

All decoding analyses were conducted using The Decoding Toolbox (TDT,Hebart et al., 2015) within MATLAB 2022 (https://www.mathworks.com/). By default, TDT utilized the “leave-one-run-out” as cross-validation. In leave-one-run-out cross-validation, the data are divided into*n*chunks, corresponding to the number of runs. During each fold or iteration, one chunk is used as the test data while the classifier is trained on the remaining chunks. The classifier’s performance is typically evaluated by averaging the classification accuracy across all folds. Since there were six runs in our experiment, all the decoding analyses were six-fold cross-validated. The beta weights derived from the GLM served as the input features for these decoding analyses.

#### 2.8.1 ROI cross-classification analysis

An L2-norm support vector machine (SVM) classifier, as the default classifier in TDT, was trained to distinguish between brain activation patterns when subjects heard the same vs. different melodies, which were defined by non-transposed-same and non-transposed-different probe melodies. Subsequently, it was further tested to determine if the distinctions could also apply on the conditions of transposition, using transposed-same and transposed-different probe melodies.

Consequently, the selection of ROIs focused on brain regions associated with melody perception, especially those identified in previous research using melody discrimination tasks (e.g.,Brown & Martinez, 2007;Foster & Zatorre, 2010;Jerde et al., 2011). Previous findings indicated that melody discrimination tasks involve working memory for melodies and the potential for participants to mentally rehearse during the process (Brown & Martinez, 2007;Janata, 2015;Jerde et al., 2011). To elaborate, melody-related task requires an active memory component, and the recruitment of BA 6 reflects the motor component of a rehearsal mechanism (Janata, 2015). Melody tasks requiring working memory also activated more in BA 6 (the precentral gyrus) than the passively listening to melody in a PET study done byJerde et al. (2011). Also, melody discrimination tasks might lead to strong activations in the precentral gyrus, which are the areas involved in vocal planning of the melodies (Brown & Martinez, 2007).

Therefore, in addition to the primary regions of melody perception such as Heshl’s gyrus (HG) and superior temporal gyrus (STG) (Koelsch, 2011;Patterson et al., 2002;Peretz & Zatorre, 2005;Schindler et al., 2013;Stewart et al., 2008), we also included regions related to melody working memory and vocal planning such as inferior frontal gyrus (IFG), inferior parietal lobule (IPL) (Foster & Zatorre, 2010;Lee et al, 2011;Levintin & Menon, 2003;Kim et al., 2020;Maess et al., 2001;Royal et al., 2016;Tillmann et al., 2006), and precentral gyrus (PreCG/BA4/BA6) (Brown & Martinez, 2007;Janata, 2015;Jerde et al., 2011) to see if the melody discrimination related processing can tolerate transposition. Consequently, these 14 ROIs encompassed bilateral HG, STG, the orbital, triangular, and opercular parts of IFG, IPL, and PreCG. These regions were delineated using the Automated Anatomical Labeling (AAL) atlas, facilitated by MarsBaR (https://marsbar-toolbox.github.io/).

To identify regions recognizing transposed-same melodies as non-transposed-same following the original melodies, the true positive rate (TPR), which represented the rate of classifying Pts as Pos, was observed. Right-tailed one-sample t-tests were then conducted to identify which of the 14 ROIs had a TPR higher than chance (chance level = 50%). To control the increasing error rates when conducting multiple comparisons, we used Benjamini-Hochberg procedure to control the false discovery rate. Meanwhile, to control the possibility that the classifier was categorizing all transposed conditions as Pos, the false positive rate (FPR) was also examined. Only the regions with significantly higher TPRs and non-significant FPRs would be included in the final results.

Additionally, a correlation analysis with behavioral performances was implemented to explore associations between the classifier performance in these regions and individual differences in their behavioral performance. These analyses sought to elucidate the neural tolerance to melody transposition and its relation to the corresponding behavioral measures. The same multiple comparison correction methods as mentioned in the last paragraph were also employed.

#### 2.8.2 Whole-brain searchlight cross-classification analysis

To further investigate, a whole-brain searchlight analysis was conducted to identify regions capable of classifying same and different conditions irrespective of transposition. The same cross-classifier was both trained and tested, utilizing a searchlight radius of four voxels spherically. The classifier’s performances were assessed in six iterations, and the results were averaged to represent accuracy at each center voxel within the searchlight. These accuracy values were then saved in a brain map for each individual.

All individuals’ brain maps underwent smoothing with an 8 mm full-width half-maximum Gaussian kernel and were subsequently aggregated for a group-level t-test. The surviving pattern after applying thresholds (voxel-wise uncorrected*p*< .001 and cluster size k >= 10) could reveal brain regions exhibiting tolerance to melody transposition. After extracting the parameter estimates within those surviving regions, we conducted a correlation analysis to explore the relationships between the observed classifier performance in these regions and individual differences in their behavioral performance of transposed melody discrimination. These analyses aimed to more exploratively pinpoint the regions showing tolerance to melody transposition, and clarify the relationship between the neural tolerance to the behavioral RP performance.

## 3 Results

### 3.1 Behavioral results

#### 3.1.1 AP performance

The result of the AP test was illustrated inFigure 2. Only two of the musicians achieved a score higher than 0.9, the threshold set byAruffo et al. (2014)to denote AP possession. Consequently, it should be acknowledged that the majority of participants did not demonstrate AP ability, and this consideration should be taken into account in subsequent analyses. Musicians (M) exhibited superior performance in the Absolute Pitch (AP) test compared to non-musicians (NM) (Welch’s*t*(10.86) = 3.39,*p*= .006).

**Fig. 2.**
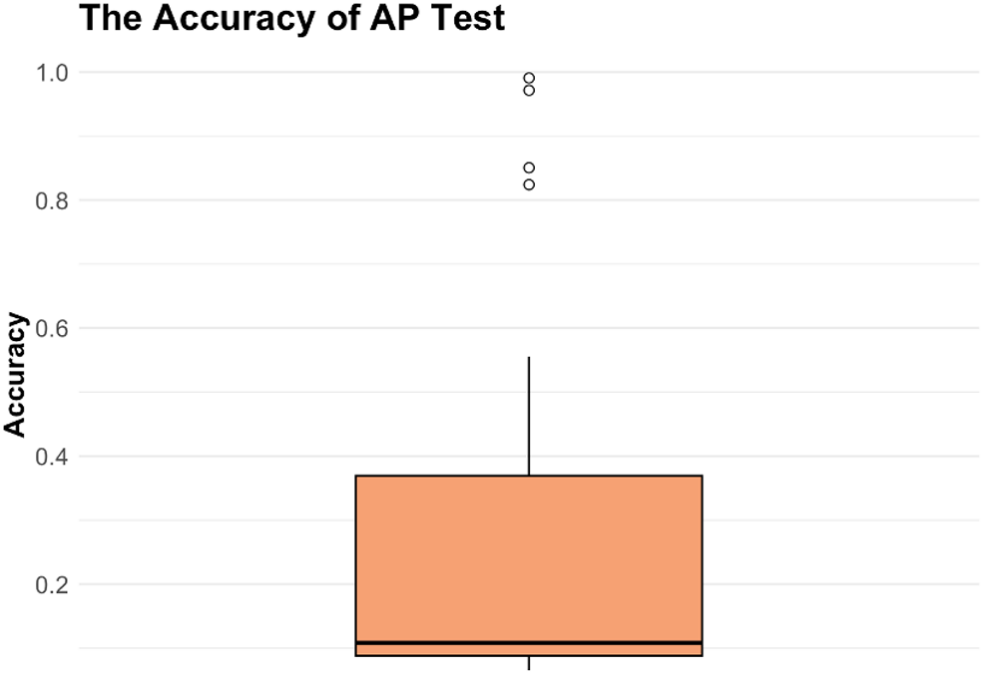
The AP score.*Notes.*Only two of the participants scored higher than 0.9, which defined the possession of AP ability.

#### 3.1.2 The different in-scanner task performances of four conditions

**Fig. 3.**
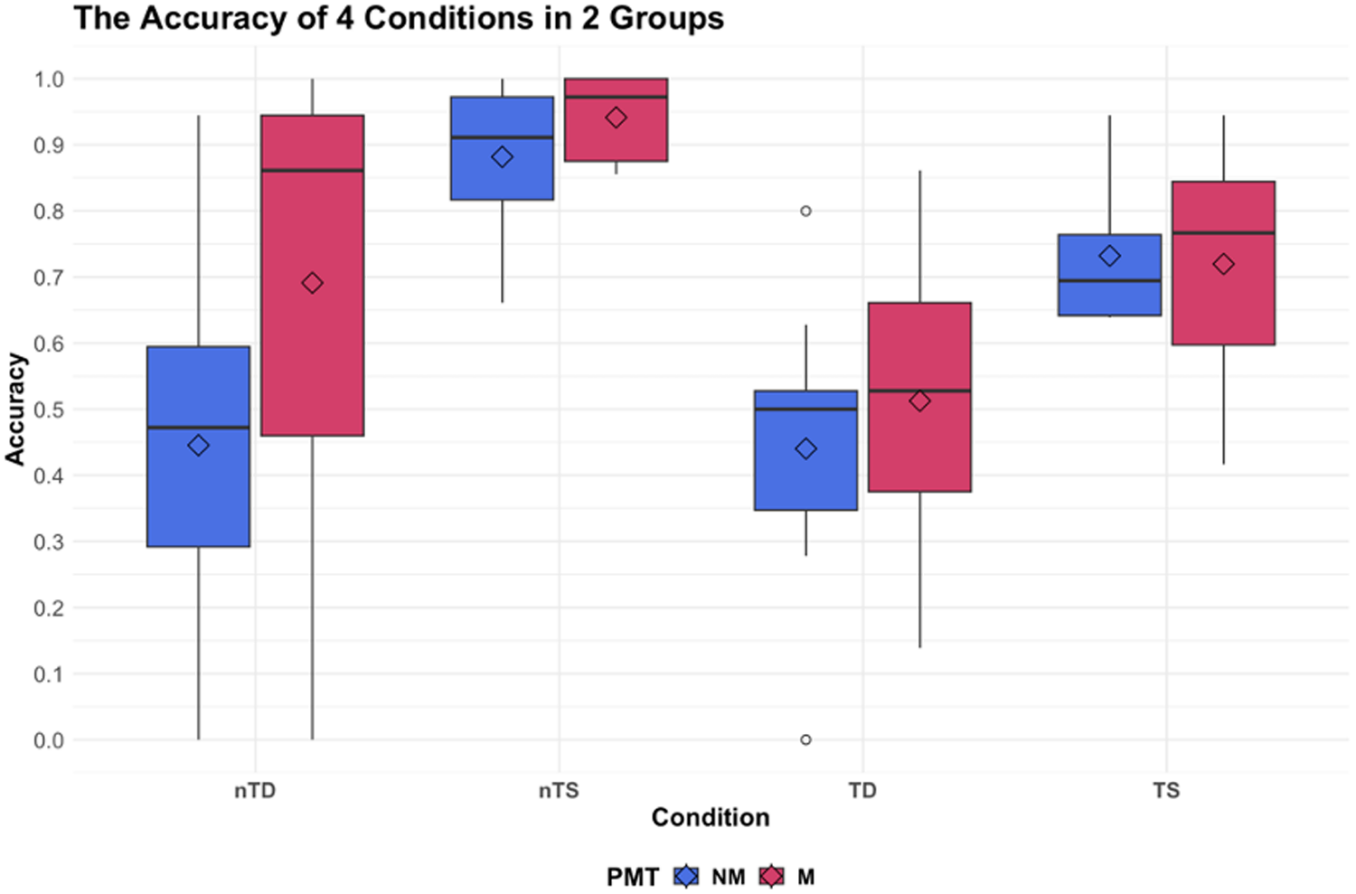
Melody discrimination task performance of musicians and non-musicians.*Notes.*nTD, non-transposed different; nTS, non-transposed same; TD, transposed-different; TS, transposed-same; PMT, professional musical training; NM, non-musicians; M, musicians.

The accuracies of the in-scanner melody discrimination task were subjected to a 2 x 4 mixed-design ANOVA. The analysis revealed a statistically significant main effect of condition (*F*(3,60) = 19.27,*p*< 10^-8^), while neither professional musical training nor the interaction between the two factors reached significance (Fig. 3). Holm-Bonferroni’s post hoc tests revealed that the accuracy was significantly higher in non-transposed same (nTS) compared to all other conditions (TS,*p*= .014; nTD,*p*< 10^-5^; TD,*p*< 10^-8^), and significantly higher in transposed same (TS) compared to different conditions (nTD,*p*= .031; TD,*p*< .001). However, there was no significant difference between TD and non-transposed different (nTD) (*p*= .154). These findings indicate that the performances across the four conditions were distinct, with nTS displaying the highest accuracy and TD showing the lowest.

#### 3.1.3 The positive correlation between RP and AP performance

Regarding the performance of transposed melody discrimination (RP performance), the accuracies of TS and TD conditions were combined and averaged. The performance of musicians and non-musicians did not show a significant difference (*t*(20) = 0.75,*p*= .46). Lastly, the correlation between AP score and RP performance was significantly positive (seeFig. 4,*r*= .58,*p*= .0045), which somewhat contrasts with the findings ofMiyazaki (2004).

**Fig. 4.**
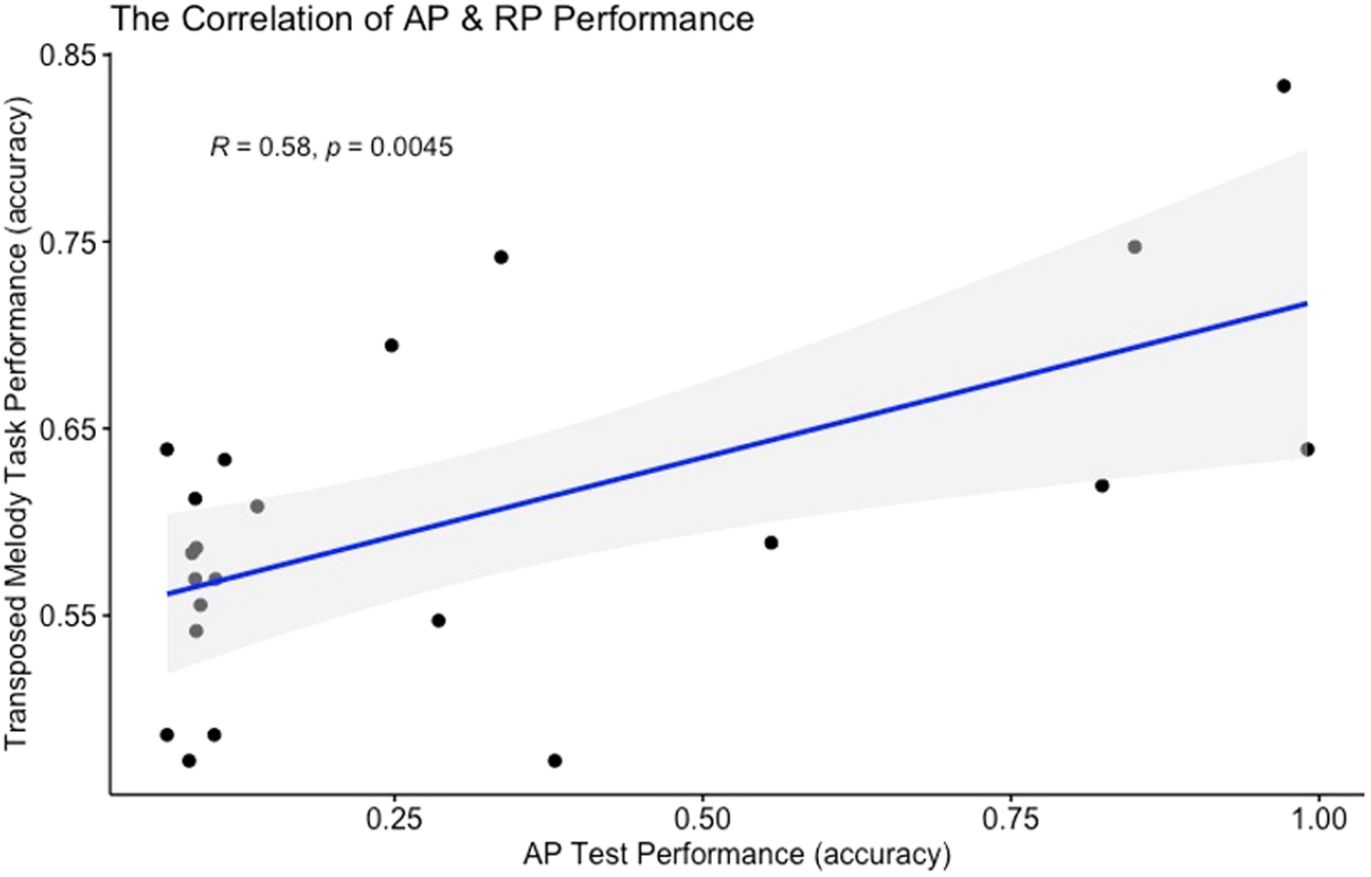
The correlation of AP and RP performance.*Note.*AP, absolute pitch; RP, relative pitch.

### 3.2 Univariate adaptation analysis

#### 3.2.1 The melody-specific adaptation effect in the auditory cortex

A contrast of the adaptation of non-transposed same (nTS) and transposed same (TS), that is, (O_prePos_– Pos) + (O_prePts_– Pts), were calculated, and examined while exclusively masked by the “uninterested adaptation” contrast-based mask, that is, (O_prePod_– Pod) + (O_prePtd_– Ptd), to isolate the melody-specific adaptation effect.

Using a loose threshold (*p*< .001 [unc.], k >= 10), activations in the right superior temporal gyrus (STG) and the left middle temporal gyrus (MTG) were identified under the contrast of interest (Table 3). Extraction of parameter estimates from this cluster revealed relatively more decreases in nTS and TS compared to nTD and TD, as depicted inFigure 5. This finding suggests a larger degree of adaptation when participants listened to the same melodies in both original and transposed keys.

**Table 3.** Peak BOLD effects showing melody-specific adaptation.

(O _prePos_ – Pos) + (O _prePts_ – Pts), exclusively masked by [(O _prePod_ – Pod) + (O _prePtd_ – Ptd)]					
Location	k	t	x	y	z (mm)
R. STG	30	4.11	60	-18	-2
		3.78	60	-26	0
		3.45	54	-32	6
L. MTG	30	3.76	-62	-26	2

*Note.*L., left; R., right; STG, superior temporal gyrus; MTG, middle temporal gyrus. Threshold at*p*< .001 (unc.) and cluster size k >= 10.

**Fig. 5.**
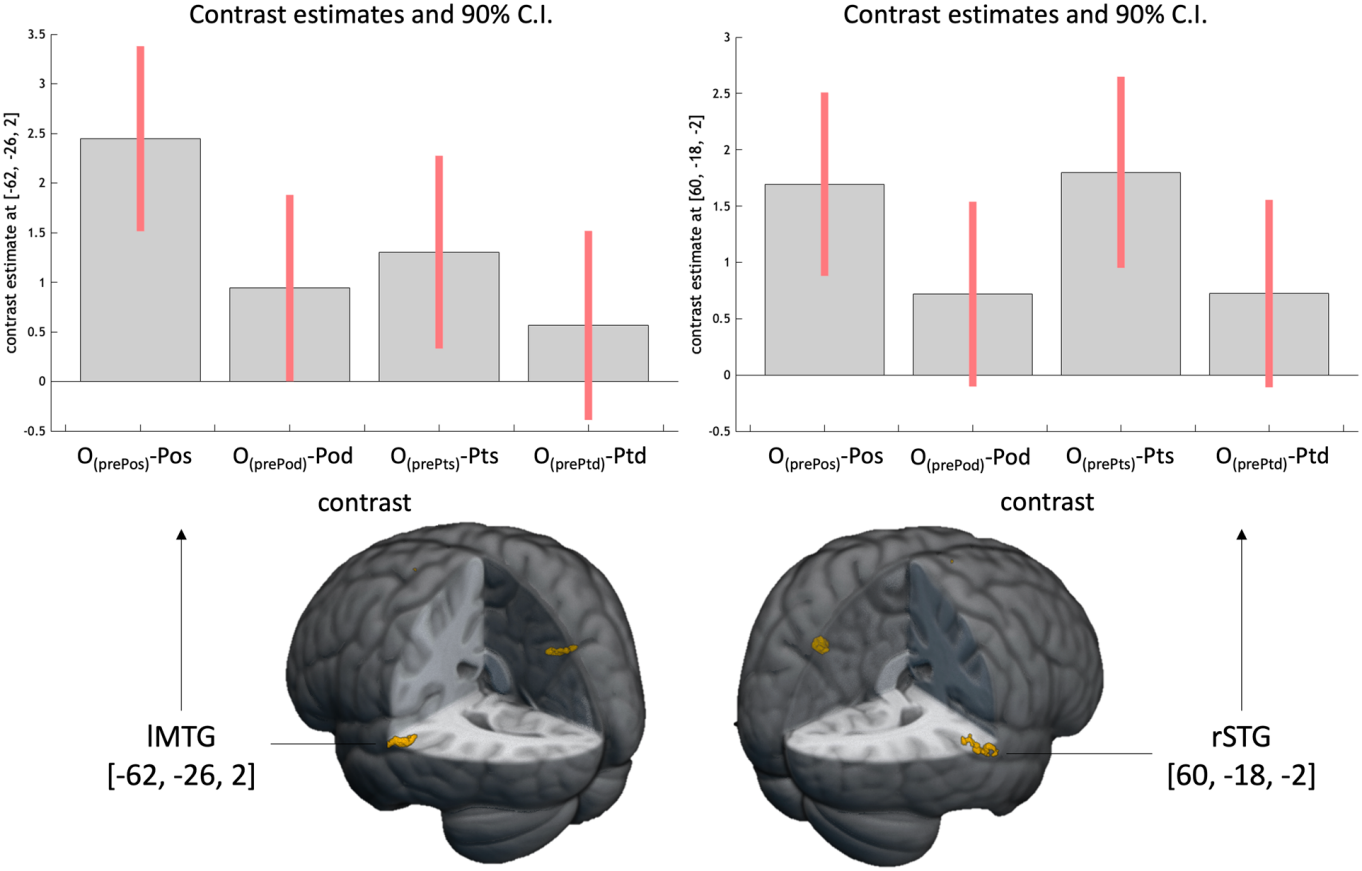
The contrast estimates of four adaptation effects in lMTG and rSTG.*Note.*The top panel of the figure shows the contrast estimates of (O_(prePos)_– Pos), (O_(prePts)_– Pts), (O_(prePod)_– Pod) and (O_(prePtd)_– Ptd) at the MNI coordinates of 1) [-62, -26, 2], shown in the bottom left; and 2) [60, -18, -2], shown in the bottom right.

#### 3.2.2 No correlation with RP performance

The contrast estimates of the melody-specific adaptation, that is, [(O_(prePos)_– Pos) + (O_(prePts)_– Pts)] – [(O_(prePod)_– Pod) + (O_(prePtd)_– Ptd)], in both clusters were calculated to do a correlation analysis with RP performance. No significant correlation was observed in both clusters (rSTG:*r*= .113,*p*= .62; lMTG:*r*= .059,*p*= .79), failing to provide evidence for the relation of the neural melody-specific adaptation and the behavioral RP performance.

### 3.3 Multivariate cross-classification (MVCC) analysis

#### 3.3.1 ROI cross-classification analysis

A linear classifier was trained to discriminate between Pos and Pod conditions and subsequently tested on Pts vs. Ptd conditions to identify regions tolerant to transposition. To identify regions recognizing transposed-same melodies as non-transposed-same following the original melodies, the true positive rate (TPR), which represented the rate of classifying Pts as Pos, was observed. Meanwhile, to control the possibility that the classifier was categorizing all transposed conditions as Pos, the false positive rate (FPR) was also examined. The decoding results within the 14 predefined bilateral ROIs are presented inFigure 6. The findings indicate that activation patterns in bilateral IFG_triangular_, IFG_opercular_, PreCG, the left IFG_orbital_, IPL, and the right STG could significantly categorize Pts as Pos above chance. However, the FPRs in the right IFG_triangular_and IFG_opercular_were also significantly above chance, which suggest that these two brain regions may lack sufficient discriminative power after melody transposition. We concluded that only the regions with significantly higher TPRs and non-significant FPRs exhibited a degree of tolerance to transposition when distinguishing between same and different probe melodies. Please note that although these regions’ FPRs are not significantly higher than 50%, they are close to their respective TPRs. One possible interpretation is that the participants might not recognize the difference occurred in Ptd and perceive Ptd as Pos, therefore leading to these relatively high FPRs.

**Fig. 6.**
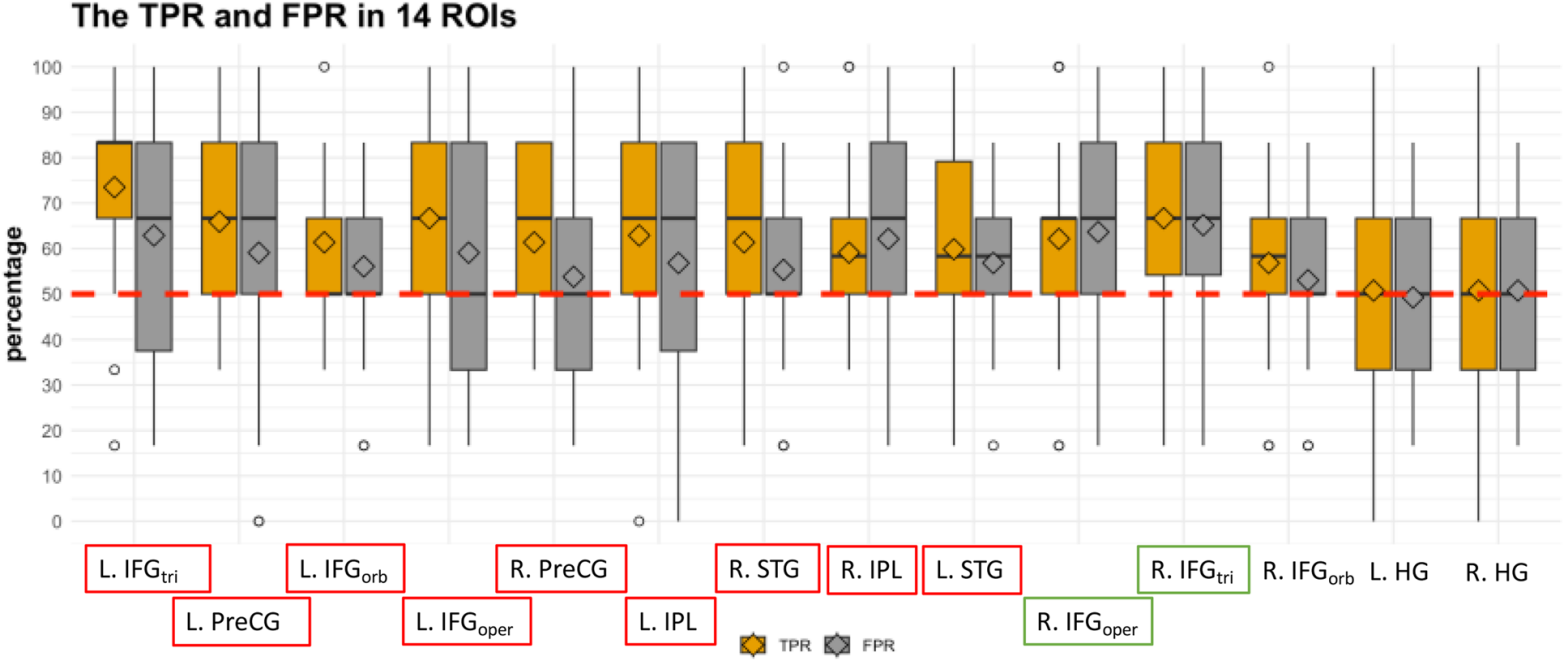
The cross-classification TPRs and FPRs in 14 ROIs.*Notes.*The brain regions within the red boxes are those where only TPR was significantly higher than chance, indicating that these regions exhibit tolerance to transposition. The two regions within the green boxes, although their TPRs were significantly higher than chance, also had FPRs significantly above 50%. Therefore, these two regions may lack sufficient discriminative power after melodic transposition.

##### 3.3.1.1 There were no correlations with RP performance

Despite the success in cross-classifying Pts as Pos in these regions, no significant correlations were found between the decoding results of these ROIs and RP performances, lack of supporting the relationship between the neural mechanisms underlying the tolerance to transposition, as revealed by the decoding analyses and the behavioral RP performance.

#### 3.3.2 Whole-brain searchlight cross-classification analysis

To delve deeper into the investigation, we employed a whole-brain searchlight classifier, training it on Pos vs. Pod and subsequently testing it on Pts vs. Ptd. Individual brain maps were aggregated to calculate the group effect, utilizing a threshold at voxel-wise uncorrected*p*< .001 and a cluster size >= 10. The results are summarized inTable 4and illustrated inFigure 7.

**Table 4.** Peak decoding accuracy of cross-classification.

Location	k	t	x	y	z (mm)
R. PreCG	124	4.84	42	-8	54
		4.26	46	-4	44
		3.76	50	4	34
White matter in right frontal lobe	56	4.68	30	-10	38
R. AG	71	4.6	32	-56	46
White matter in left frontal lobe	32	4.43	-18	-30	28
Lateral Globus Pallidus	15	3.96	-18	-4	8
R. STG	10	3.94	54	-24	4
L. PreCG	12	3.72	-26	-4	48

*Notes.*R. right; L. left; PreCG, precentral gyrus; AG, angular gyrus; STG, superior temporal gyrus. Threshold at*p*< .001 (unc.) and cluster size >= 10. Masked with an ICV mask.

**Fig. 7.**
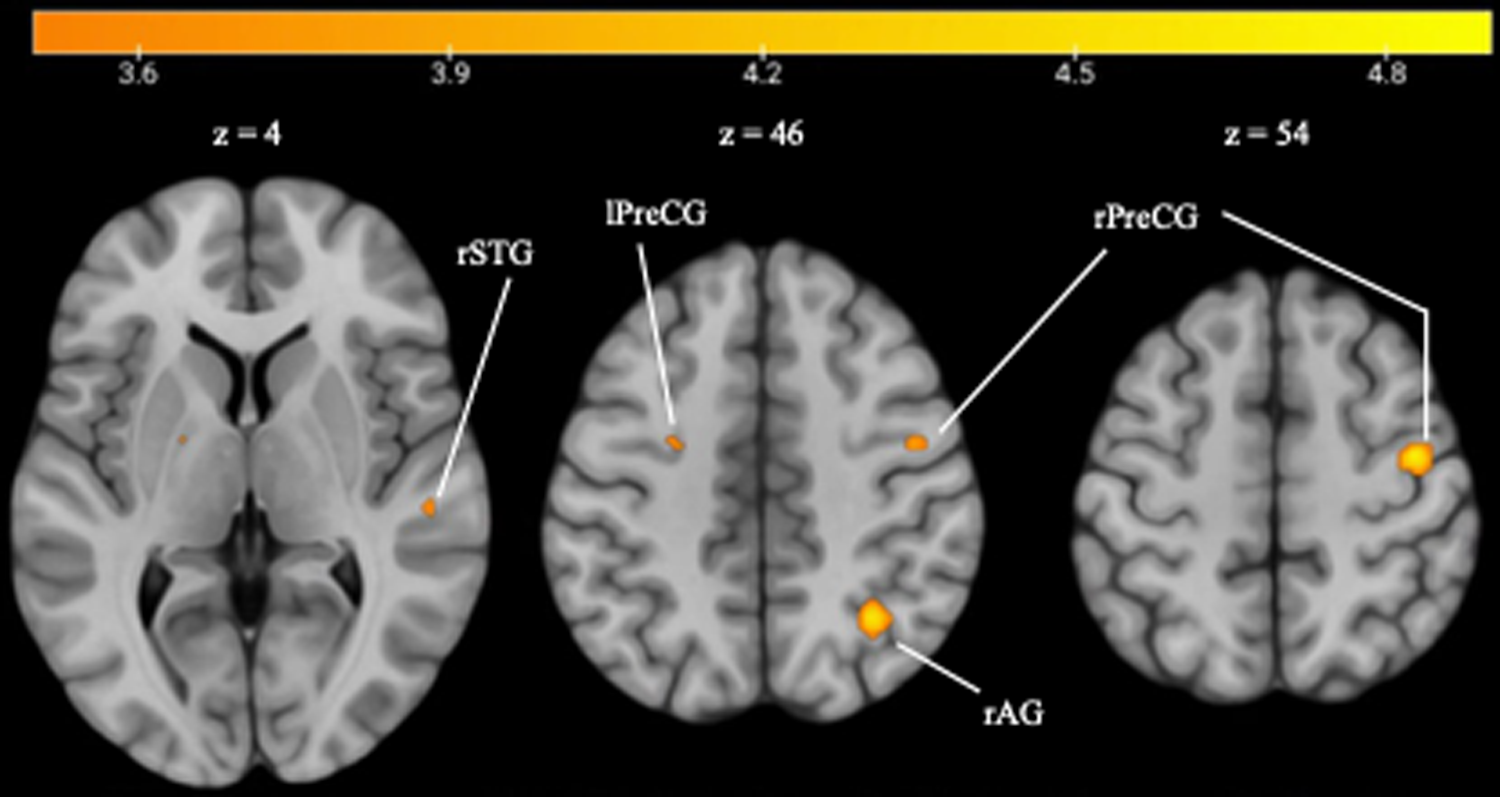
Searchlight MVCC results.*Notes.*rSTG, right superior temporal gyrus; lPreCG, left precentral gyrus; rAG, right angular gyrus; rPreCG, right precentral gyrus. Threshold at*p*< .001 (unc.) and cluster size >= 10.

The findings indicate that bilateral PreCG, the right angular gyrus (AG), and the right STG exhibited above-chance decoding accuracy. This implies that the activation patterns in these regions could effectively discriminate between same and different probe melodies regardless of transposition. This result supports the results from the ROI analysis that bilateral PreCG and the right STG demonstrate tolerance to transposition, and suggests that the right AG might also potentially exhibit tolerance to melody transposition.

##### 3.3.2.1 There were no correlations with RP performance

Similar to the previous ROI analysis, we failed to detect any significant correlations between the decoding results and the behavioral RP performance.

## 4 Discussion

In the present study, we examined the neural processing of melodies with a specific focus on tolerance to musical transposition. Employing an adaptation paradigm, we identified clusters in the right STG and the left MTG that exhibited heightened adaptation when consecutive melodies were identical, irrespective of transposition, compared to when they differed. Additionally, the MVCC analysis revealed that activation patterns in the bilateral PreCG, the left IFG, the left IPL, the right AG, and the right STG demonstrated tolerance to melody transposition when discerning the similarity or dissimilarity of melodies from their original counterparts. Notably, neither the melody-specific adaptation effect nor the cross-classification performance exhibited correlations with the participants’ relative pitch behavioral performance.

The melody discrimination task necessitated participants to memorize, rehearse, and manipulate the original melodies for comparison with subsequent ones. Consequently, the observed activation patterns were not limited to auditory perception but also implicated working memory and cognitive processes related to motion. Our findings provide compelling evidence supporting the existence of melody transposition tolerance along the neural processing pathway, spanning temporal, parietal, and frontal regions.

Unexpectedly, our results did not reveal any transposition tolerance in the primary auditory cortex, Heschl’s gyrus (HG). This finding contradicts previous assertions bySchindler et al. (2013)that melodies are invariantly represented in the primary auditory cortex. Lateral HG has been implicated in pitch processing in prior studies (Kim et al., 2020;Patterson et al., 2002;Schneider et al., 2005). Based on the hierarchy of melody processing proposed byPatterson et al. (2002), HG is responsible for short-term pitch processing, while the more anteriolateral STG and planum polare (PP) are involved in determining pitch changes in melodies. Therefore, in the current case of using various melodic stimuli, HG might not represent the invariance property of melody.

The absence of a discernible relationship between RP behavioral performance and neural tolerance to transposition was unexpected. Despite the absence of literature supporting such a correlation, initial intuition led us to predict a positive association. However, neither the melody-specific adaptation effect nor cross-classification performance exhibited correlations with RP performance. We posit that the elevated difficulty of the “different” conditions may account for these results, with particularly low behavioral performance observed in the transposed different (TD) condition. This suggests a potential confounding factor in the calculation of the melody-specific adaptation effect. Additionally, the task’s high difficulty level influenced its discriminant validity, resulting in consistently poor RP performances. Both the potential confounding factor and overall poor performance may contribute to the non-significant results obtained.

To address a possible concern about the sample size in our study being too small, we referred to several relevant past studies. Our experimental design primarily referencedFoster and Zatorre (2010), who recruited 20 participants. Although they divided participants into musician and non-musician groups, the study’s main focus was on within-subject comparisons, aiming to identify the neural substrates of relative pitch. This study used univariate analysis to compare the differences when melodies were transposed and not transposed, and obtained the significant results. Therefore, recruiting 22 participants for our study with a similar design should be sufficient to compare univariate within-subject differences.

Additionally, we conducted MVPA, including both ROI and searchlight analyses. The most relevant study to ours isSchindler et al. (2013), which aimed to identify Gestalt representations of melodies in the human primary auditory cortex. They trained a classifier to distinguish between original melodies one and two, and then tested it to differentiate transposed versions of these melodies. They successfully identified regions in the primary auditory cortex that could accurately distinguish transposed melodies above chance level. Although they only recruited eight participants, this study still provided the first direct evidence of relative pitch information representation in the primary auditory cortex. To conclude, we believe that recruiting 22 participants for this study is sufficient to draw robust findings on within-subject comparisons.

In summary, our findings propose a neural basis for melody transposition invariance, specifically indicating tolerance to transposition during melodic information processing. However, the extent and nature of this transposition invariance across different stages of melody processing remain unclear. Despite these uncertainties, this study sheds light on potential brain regions displaying tolerance to melody transposition, contributing to our understanding of the intricate neural mechanisms underlying musical perception.

## Data Availability

All the scripts and the data (melody stimuli files as well as imaging data) are available via a request to the corresponding authors with a data sharing agreement.
